# *“I just feel alone and by myself”:* how adolescents experience loneliness when their parent has cancer

**DOI:** 10.1186/s12889-026-27112-x

**Published:** 2026-03-31

**Authors:** Lydia Mckeown, Martin Dempster, Jenny Groarke, Lisa Graham-Wisener

**Affiliations:** 1 School of Psychology, Centre for Improving Health‐Related Quality of Life, Queen’s University Belfast, Belfast, UK; 2 School of Psychology, National University of Ireland, Galway, Ireland

**Keywords:** Parental Cancer, Loneliness, Youth Mental Health

## Abstract

**Background:**

Parental cancer is a distressing experience for young people which is associated with an increased risk of behavioural and emotional issues as well as depression and anxiety. Systematic review evidence indicates that young people experiencing parental cancer report profound feelings of loneliness. Despite this evidence, there is a lack of dedicated focus given to adolescent loneliness when a parent has cancer. This study aimed to better understand adolescent’s subjective lived experience of loneliness when their parent has cancer and considers factors which might influence their experience.

**Methods:**

Nine qualitative interviews were conducted with young people aged 18 to 19 years old who self-identified as lonely when their parent had cancer. The sample included four women and five men. The interviews were audio-recorded, transcribed verbatim, and analysed using reflexive thematic analysis.

**Results:**

Young people reported several facets of loneliness including temporal loneliness, affective loneliness, relational loneliness, existential loneliness and feeling like a changed person as a result of parental cancer. Young people’s loneliness was exacerbated by constrained communication with their parent and missing who their parent was prior to cancer. Normal family life had to shrink around the parent’s cancer diagnosis which left young people feeling lonely. Young people hid the pain of parental cancer from their friends. Even in cases where young people were keen to socialise, caring responsibilities limited their time to do so. Young people reported that discussing parental cancer with peers with similar lived experience and socialising with selected trusted friends was helpful when their parent had cancer. Loneliness could also be alleviated by getting support from trusted adults and techniques such as journalling to support emotional processing and catharsis.

**Conclusions:**

Adolescents experiencing parental cancer report intrapersonal loneliness and interpersonal loneliness across their peer group and family life. Healthcare professionals should identify if patients have young dependent children early on so they can support parents to provide age-appropriate information about cancer to their young people and signpost parents to relevant support for their children. This study has provided a strong foundation to begin developing a theoretical model of loneliness in adolescents experiencing parental cancer.

**Supplementary Information:**

The online version contains supplementary material available at 10.1186/s12889-026-27112-x.

## Background

Parental cancer is a distressing experience for young people which is associated with an increased risk of behavioural and emotional issues [24, 35]. Young people experiencing parental cancer report clinically significant post-traumatic stress symptoms, depression and anxiety [40]. Despite this, little is known about loneliness when a parent has cancer. The findings of our recent qualitative systematic review [31] suggest that loneliness is central to the experience of parental cancer and this cohort should be interviewed directly about loneliness. Increasingly, there is acknowledgement in the wider literature that cancer patients experience ‘cancer-related loneliness’, proposed to be similar yet distinct from loneliness experienced by the general population [12, 14]. This suggests that adolescents’ experiences of loneliness when a parent has cancer might also present unique challenges. The understanding gained from the current study is essential to support the development of a theoretical model of loneliness in adolescents experiencing parental cancer, with the view of optimising existing services, and developing novel interventions to better support this group of youth.

Adolescence is a sensitive developmental period that takes place aged 10 to19 years old (World Health Organisation, n.d.). For many young people, adolescence is a time of loneliness. Loneliness is a negative emotional experience resulting from the perception that an individual's social network is lacking in quality or quantity [36]. Compared to less than 20% of younger children, over 50% of adolescents report recurrent loneliness [22, 37]. Loneliness is also prevalent among university aged adolescents, with 92% of university students reporting loneliness [41]. In youth, loneliness is linked to a range of mental health issues such as suicidal ideation, depression and anxiety [27, 43]. The impact of loneliness during adolescence can have long-term consequences, including earning less in midlife [52], and an increased risk of depression in adulthood [19].

Findings from a quantitative longitudinal study [18] which explored adolescents’ emotional reactions to parental cancer, indicated that loneliness was associated with emotional and behavioural problems. Although helplessness and uncertainty decreased over the first year of the cancer diagnosis, loneliness remained stable [18]. Qualitative research indicates that this group of youth feel excluded from their parent’s medical treatment and feel emotionally detached from others when their parent has cancer [26]. Our qualitative systematic review [31] concluded that young people report feeling profoundly lonely when their parent had cancer. Factorssuch as peers not understanding their lived experience, changed family dynamics and constrained communication at home intensified loneliness.Ourreview [31] recommended that future research should interview this population of young people directly, to develop a more comprehensive understanding of their lived experience. This is aligned to recent policy recommendations by the UK government’s ‘*Tackling Loneliness Evidence Review’* [47]*.* The review emphasised a need to consider loneliness in smaller subgroups of youth and to prioritise in-depth qualitative work. The current study also addresses research priorities indicated by cancer patients themselves in *The James Lind Alliance Priority Setting Partnership* [33]. The exercise highlighted a need to explore the impact of parental cancer and how best to support dependent young people. To our knowledge, this is the first qualitative study worldwide to place a dedicated focus on how adolescents experience loneliness when their parent has cancer. This study aims to better understand adolescents’ subjective lived experience of loneliness when their parent has cancer and considers factors which might influence their experience.

### Methodology

#### Design

This study adopted a qualitative semi-structured interview approach. We aimed to explore how people described loneliness and what factors influenced their experience. Loneliness in this cohort is under researched, meaning an exploratory qualitative approach was essential to gain an in-depth understanding of their lived experience. Loneliness is also considered an inherently subjective experience which is likely experienced differently by everyone [29]. With this understanding in mind, quantitative methods are unlikely to capture the full complexity of loneliness in this cohort. This study adopted an experiential qualitative framework informed by Reflexive Thematic Analysis (RTA; [9]). RTA is used when exploring participants’ personal lived experiences [7]. It is also flexible in nature and allows for the identification of underlying themes, beyond what is clear from the data on a surface level [7]. This approach allowed the authors to explore commonalities and idiosyncrasies across participants’ accounts of loneliness. This supported a more developed understanding of adolescent loneliness when a parent has cancer.

We adopted RTA within a critical realist epistemological stance, to explore participants lived experience of loneliness when a parent has cancer, using their own definitions. Critical realism proposes that a reality exists which is independent of our perception, yet our knowledge of this is mediated by theory and context [17]. This understanding allowed the research team to focus on the young peoples lived experience, while also appreciating how their experience may be influenced by contextual and structural factors [17]. The use of a critical realism epistemological framework is appropriate due to RTA’s theoretical flexibility [5, 8].

### Ethical approval

Ethical approval for this study was obtained prior to data collection. The study followed ethical guidelines by the British Psychological Society and the Declaration of Helsinki (2013). All participants took part on a voluntary basis.

### Identification and recruitment

Participants were recruited via purposeful sampling criteria to include:

i) experience of loneliness based on the participants own definition of loneliness
ii) ii) experience of parental cancer treated with curative intent, between the ages of 10 and 19 years old. (Curative intent refers to treatment which aims to hopefully remove/destroy all cancer)
iii) iii) currently aged 10 to 19 years old
iv) iv) themselves and their parental figure were currently living in the UK at the time of the interview.

There is no fixed sample size outlined for RTA. However, it is advised that the sample should be simultaneously large enough to allow a thorough comprehension of the phenomenon being studied and small enough to enable an in-depth and case-oriented analysis [49]. Nine participants were recruited for this study which provided sufficient information power according to Malterud et al. [28]. Participants gave a rich account of experiences and provided high quality interview dialogue across the nine interviews [28].

The research team contacted third sector organisations providing support for individuals with cancer and their families in the UK to share recruitment materials with them. Social media recruitment was utilised such as advertising in cancer support groups on Facebook.. Two participants were recruited from a university as the study was advertised to first year psychology students. Participants who took part were awarded a £20 amazon voucher and the psychology students also received course credit.. As all participants were 18–19 years old, they provided full informed consent for themselves.

### Data collection

Semi-structured interviews with nine young people aged 18–19 years old were conducted by the researcher LM via Microsoft Teams between March 2024 and February 2025. The interviews lasted 15 min to 25 min, with an average time of 19 min. In addition to online written consent, verbal consent was sought and recorded before each interview. Only the researcher and the participant were present during the interview. LM conducted this study during her PhD studies. She had substantial training in qualitative methods before completing the study. There had been no previous contact between the researcher and participants prior to the study. A broad topic guide was developed to include open-ended questions and probes and was reviewed by Professor Pamela Qualter, a youth loneliness expert (Please see Supplementary material 1: Topic Guide). This guide was also reviewed and refined with input from a public and patient involvement panel (PPI) of three young adults who experienced parental cancer during their adolescent years. Interviews were audio-recorded and transcribed verbatim using Mac Whisper software and then thoroughly checked for accuracy. After taking part in the interview, participants were provided with a debrief sheet with details of available support.

### Data analysis

The interview transcripts were analysed using reflexive thematic analysis (RTA). Transcripts were imported to NVivo to organize the data and facilitate analysis. The iterative six-stage reflexive thematic analysis (RTA) process outlined by Braun and Clarke [4, 6] was followed. RTA involves (i) repeated readings of interview transcripts to become familiar with the data, (ii) coding units of relevant information related to the aims of the study, and (iii) categorising these codes into initial themes. The first three stages were carried out by LM who adopted an inductive semantic and latent driven approach. LM lead the remaining stages of RTA, (iv) reviewing the themes, (v) defining and naming themes, and (vi) producing the final report. The final three steps were reviewed with the broader team (LGW, MD and JG).

### Reflexivity

In this study, I approached the research from an ‘outsider’ position [15] as I have no experience of a parent having cancer. Although I was aware of my outsider status prior to my interviews with young people, I became more conscious of it during interviews as many participants asked me if I had personal experience of parental cancer.. I was aware that research with this group of young people was very sensitive and as I was an outsider, I needed to ensure any questions I asked were suitable. Throughout the research process I engaged with a Patient and Public Involvement Advisory Group of young adults whose parent had cancer when they were an adolescent. The members offered suggestions on how to approach interviews sensitively. The analysis of the data from these interviews was likely influenced by the research teams disciplinary training in psychology. Preliminary themes were developed by LM who presented these themes to the research team. Through conversation themes were refined and reviewed to complete the final report.

### Validity and rigour

This study is reported in line with The Reflexive Thematic Analysis Reporting Guidelines [9]and The Quality Criteria: General and Specific Guidelines for Qualitative Approaches in Psychology Research [11]. This report provides a transparent account of the methodological and analysis procedures and was pre-registered on Open Science Framework. The researcher has acknowledged their influence on the data through detailing their reflexive practice, theoretical assumptions and epistemological positionality. Richness of the data is conveyed through illustrative quotes. This study contains all qualities that are required for high-quality reflexive thematic analysis research and ethical principles were upheld throughout the research [9, 11].

### Findings

Nine young people aged 18–19 years old and living in the United Kingdom were interviewed (see Table 1 for participant characteristics). Five of the young people’s parents had previously been diagnosed with cancer and completed radiotherapy or chemotherapy by the time of the interview. Four young people’s parents were currently diagnosed with cancer and still were undergoing radiotherapy or chemotherapy when the young person was interviewed. All young people self-identified as ‘lonely’ based on their own definition.

**Table 1:** Participant socio-demographic characteristics (*n* = 9)

**Age**	Mean age = 18.6 years old, Standard Deviation = 0.53
Gender	Women: *n* = 4(44%) Men: *n* = 5 (56%)
Race	Black African: *n* = 4(44%) Black Caribbean: *n* = 3 (33%) White: *n* = 2 (22%)
Country	England: *n* = 6 (67%) Scotland: *n* = 1 (11%) Northern Ireland: *n* = 2 (22%)
Education/Employment Status	At school/college/University: *n* = 9 (100%)
Parent With Cancer	Mother: *n* = 4 (44%) Father: *n* = 5 (56%)
Type of Cancer	Breast Cancer: *n* = 3 (33%) Lung Cancer: *n* = 1 (11%) Small Cell Lung Cancer: *n* = 1 (11%) Kidney Cancer: *n* = 1 (11%) Pancreatic Cancer: *n* = 1 (11%) Prostate Cancer: *n* = 1 (11%) Bladder Cancer: *n* = 1 (11%)

The reflexive thematic analysis resulted in four themes and sixteen subthemes (Please see Table 2 for a Coding Tree of Themes and Illustrative Quotations). Theme 1 discusses facets of loneliness when a parent has cancer, including temporal loneliness; affective loneliness; relational loneliness; existential loneliness and feeling like a changed person. Theme 2 discusses loneliness within family life when a parent has cancer, including issues such as not wanting to burden the ill parent; feeling excluded from medical information; grieving the loss of who their parent was prior to cancer and a feeling of normal family life shrinking around cancer. Theme 3 focuses on feelings of social loneliness among peers when a parent has cancer comprised of hiding the pain of parental cancer from friends; navigating pity and reactions to the word ‘cancer’ and dealing with caring responsibilities which limit time to socialise. Finally Theme 4 focuses on protective barriers against loneliness when a parent has cancer such as relating to those with similar experiences; socialising with friends; getting support from trusted adults and the use of emotional regulation techniques.

**Table 2:** Coding tree of themes and illustrative quotations

Theme	Subthemes and Illustrative Quotation
Theme 1: Facets of Loneliness When a Parent Has Cancer	**• Temporal Loneliness:** *“…for a few hours I may not feel lonely but most of the time…I feel lonely.”* **• Affective Loneliness:** *“I had kind of like anxiety attacks… that just ran.. throughout the day… if I just think too much.. like on my own, my room…”* **• Relational Loneliness:** *“You do not have anyone around you to share your burdens with.”* **• Existential Loneliness:** “*it's being in your own world, living without the people who are there for you. Just you being out on the world in an empty space.”* **• Feeling Like a Changed Person:** *“I'm actually changing who I am in real life because my life now is something else I was not expecting.”*
Theme 2: How Loneliness Manifests Within Family Life When a Parent Has Cancer	**• Not Wanting to Burden the Ill Parent:** “*I didn't like bringing up to my mum in case it would freak her out”* **• Feeling Excluded from Important Medical Information:** *“I know she speaks… to some of her friends … I just get some self-information from them that she really wouldn't want to share with me.”* **• Grieving the Loss of Who Parent Was Before Cancer:** “*…she doesn't really like do things the way she used to do…now it's just opposite…Everything has totally changed.”* **• Normal Family Life Shrinks Around Cancer:** *“Most times I just feel my mom is not giving me the attention because her diagnosis has cost her a whole lot of her time and her attention.”*
Theme 3: Feelings of Social Loneliness Among Peers When a Parent Has Cancer	**• Hiding the Pain of Parental Cancer from Friends:** *“I wouldn't want…to tell.. because I wouldn't want to…put a damper on moods…”* **• Navigating Pity and Reactions to the Word ‘Cancer’:** *“… they are being empathetic, and I often don't really like it because it … it.. leaves me in a sombre mood.”* **• Caring Responsibilities Limit Time to Socialise:** *“My schedule was definitely…different.. in the morning my dad was going to chemo I'd have to look after the dog … I couldn't have left…even if I wanted to.”*
Theme 4: Protective Barriers Against Loneliness When a Parent Has Cancer	**• Relating to Those with Similar Experiences:** “*I met a group of kids who… had either a mum or dad who was suffering from cancer… So those were the persons I felt comfortable… talking about this whole situation with.”* **• Socialising With Friends:** *“They often visit me on weekends and when I'm less busy and try to make me enjoy the moment.”* **• Getting Support from Trusted Adults:** *“They often visit me on weekends and when I'm less busy and try to make me enjoy the moment.”* **• Emotional Regulation Techniques:** *“Yeah like I have got a journal as well, so I always write stuff down which really helps.”*

### Theme 1: facets of loneliness when a parent has cancer

This theme relates to the different types of loneliness adolescents experience when a parent has cancer. Across accounts, participants described experiences of how loneliness was experienced temporally. Participants discussed affective loneliness such as feelings of depression and anxiety. Loneliness was also described relationally, such as feeling alone amongst others. Young people described profound experiences of existential loneliness and feeling like a changed person as a result of parental cancer.

#### Subtheme 1: temporal loneliness

Young people reported that they were lonelier at particular times of the day such as the evening or when the young person was unoccupied. All participants in this study were enrolled in education meaning they were busy during the daytime focusing on their studies. Quieter t times such as the evening may trigger young people to ruminate on feeling alone and intensify their experience of loneliness. In other cases, loneliness was described as a constant feeling that persisted all day.: *“…for a few hours I may not feel lonely but most of the time…I feel lonely.”* (Liam, 18-year-old man)*.* For some young people, loneliness when a parent has cancer is an ongoing and persistent state regardless of temporal influences.

#### Subtheme 2: affective loneliness

Multiple participants described loneliness as an emotionally distressing feeling which impacted their wellbeing. participants highlighted loneliness was a build-up of unshared emotions which manifested as feelings of low mood. Loneliness was described as a discrepancy between a happy social façade presented to others and their depressed internal state: “*on the outside you might look so happy… but deep inside you're having a huge low,”* (Callum, 19-year-old man). Young people protected themselves by presenting to others as unaffected by parental cancer, while they internally navigated difficult emotions and feelings of loneliness.

Another young person noted that loneliness is a feeling of depression, indicating that there is likely an overlap between feelings of low mood and loneliness: *“Sometimes I'm really depressed, I often am depressed…”* (Henry, 18-year-old man). One participant highlighted that loneliness could cause suicidal ideation, reflecting a strong desire to escape emotionally distressing feelings: *“to feel lonely.. it's just like losing people around you… Things become irritating to you. You feel like … like committing suicide…”* (Liam, 18-year-old man). These descriptions suggest that emotional distress is central to the experience of loneliness when a parent has cancer.

Participants reported symptoms of anxiety such as rumination when their parent had cancer. In most cases the young people did not seek any social support from friends or family to help with this. Instead, during anxiety attacks the young person would isolate themselves to their bedroom to cope alone: *“Yeah, definitely, I was very scared, I think I had kind of like anxiety attacks… that just ran.. throughout the day… if I just think too much.. like on my own, in my room…”* (Eve, 19-year-old woman). Other young people worried constantly about if the parent’s cancer progressed and led to their death: *“you're having this feeling that you might wake up one morning and they are no longer there”* (Ben, 19-year-old man)*.* Although all young people who took part in this study stated that their parent’s cancer was curable, they still felt anxious about their parents’ survival. This intensified feelings of emotional loneliness.

Participants experienced feelings of separation anxiety, and their lives became increasingly enmeshed with their ill parent. Some young people in the study decided to forego or delay developmental milestones such as accepting a place at university.. One young person stated: “*I didn't want to go… if I should leave my mum… I'm not going to see her anymore. So, I just don't want that feeling. I just want to see her”* (Chloe, 19-year-old woman). Across the narratives, young peoples’ decision to isolate themselves resulted in heightened anxiety.

#### Subtheme 3: relational loneliness

Participants reported experiencing loneliness in relation to others. Several young people defined loneliness as a feeling of isolation: *“it’s just like feeling isolated”* (Amelia, 19-year-old woman)*.* Young people felt deeply alone in their experience of parental cancer, and this was intensified by experiencing physical and emotional isolation from their close persons: *“I just feel alone and by myself.”* (Henry, 18-year-old man). Some young people reported that loneliness was inherently linked to isolation they lacked close relationships: “*I don't have anyone to share with, so it affects my emotional stability a lot”* (Henry, 18-year-old man). The lack of a support network which provides an opportunity to share about parental cancer may led to an increase in internalised distress for young people and intensify loneliness.

Participants highlighted that loneliness was exacerbated by physical separation from the ill parent who was in hospital: *“One time I went to visit my dad at the hospital…I wasn't allowed to see him.”* (Helen, 18-year-old woman). some young people also felt isolated from their well parent who was accompanying their ill spouse through treatment: *“So I was just alone because my mum needed to be there…I was just alone”* (Henry, 18-year-old man). Many young people felt that parental cancer was a weight they had to carry alone and there was a lack of people around them to help them process this experience. Although loneliness and social isolation are distinct conceptually, for young people experiencing parental cancer there is an overlap in how they discuss loneliness and isolation.

#### Subtheme 4: feelings of existential loneliness

Participants reported feelings of existential loneliness and felt imprisoned within their inner world of loneliness and parental cancer. Consequently, participants were unable to connect with their usual support system which they perceived to be situated outside of their inner world. For many young people this manifested as feeling separate from others. One participant noted that: “*it's being in your own world, living without the people who are there for you. Just you being out on the world in an empty space”* (Helen, 18-year-old woman). This suggests that when a parent has cancer, young people experience feelings of separation and confinement as part of loneliness.

For some participants, feelings of existential loneliness were intensified by social constraints, such as parental cancer being kept a secret: *“we weren't sure if the cancer had spread… I did feel…like very separate from my friends…they didn't know what was going on”* (Eve, 19-year-old woman). Not sharing information about parental cancer with close persons appeared to increase young people’s perception that they are separate from others. One young person was aware that she was not literally separate from others but noted that feelings of existential loneliness were difficult to challenge: *“in my subconscious I feel like I'm not separate and like … people are going through this.. I have friends but it's just …. it is hard sometimes.”* (Amelia, 19-year-old woman)*.* Other young people reported feeling stuck in a loop of rumination when asking existential questions: *“I try to play court … why did this actually happen to my mum..? why should it be my mum…?”* (Chloe, 19-year-old woman). This young person perceived parental cancer as an injustice to her family which further deepened her sense of separation from others.

#### Subtheme 4: feeling like a changed person

Young people felt "changed" as a result of parental cancer, which indicated an alteration to the young person’s identity. Cancer was a profound disruption and shock to the young person and their family, which destroyed their normal everyday life: “*We weren’t preparing for this thing, we never expected it, so it…hit our family and shattered our sense of normalcy”* (Callum 19-year-old man).

Young people's lives were altered by the diagnosis, and they were now expected to become more self-reliant. One young person noted that they had to become “*very independent from my life before”* (Helen, 18-year-old woman)*.* This abrupt disruption to ordinary life created feelings of disconnection from the young person’s existing life narrative and previous identity. Experiences of loneliness were intensified by the young person’s life no longer fitting their expectations. Participants explicitly reported that they felt changed as a person, as result of learning of the parent’s diagnosis: *“I'm actually changing who I am in real life because my life now is something else I was not expecting.”* (Chloe, 19-year-old woman). Young people’s perception of themselves was altered as a result of this momentous change to their external world.

One young person explained that: “*my world and life was living happily and without any threat, then my dad began to experience cancerous symptoms,…it was more…up and down”* (Callum, 19-year-old man). It was evident across narratives that parental cancer was an abrupt disruption to a young person’s normal life. This led to a shift in the young person's identity and a sense of separation from their "old" self.

### Theme 2: how loneliness manifests within family life when a parent has cancer

The theme relates to how loneliness manifests within family life when a parent has cancer. Within the family home, young people experience loneliness due to not discussing their feelings to avoid burdening their parents. Similarly, parents often withhold important medical information from their children which can intensify feelings of isolation and loneliness. Young people described that their loneliness was intensified by missing their day-to-day family life prior to the parent’s cancer diagnosis.

#### Subtheme1: not wanting to burden the ill parent

Participants self-imposed strict boundaries when communicating at home and decided in most cases not to approach their ill parent with their personal problems as they felt it would be inconsiderate to ‘burden’ them. Young people reported that they enforced these self-imposed boundaries whilst their parent was receiving active treatment: “*Because my dad still has cancer, you don't really want to like add more stress on to the family as well”* (Amelia, 19-years-old).

Some participants reported that discussing cancer may have distressed their parent who was already in a vulnerable state. This might reflect the parentification of some of the young people in this study, whereby the child becomes responsible for the emotions of the parent: “*I didn't like bringing up to my mum in case it would freak her out”* (Eve, 19-year-old woman). Several young people felt that their personal life seemed insignificant to discuss when their parent was dealing with cancer: *“They have more things to worry about other than my own personal life”* (Henry, 18-year-old man). These self-inflicted limitations on communication with the ill parent often led to the young person dealing with negative emotions alone: *“So, whenever I know, I am…going through a lot.. I don't try to… discuss things with her…I don't want to cause more trouble”* (Chloe, 19-year-old woman).

Feeling less able to confide in the parent for fear of upsetting them was particularly hard for young people who would have sought advice from their parent before cancer: *“I used to tell my mom a lot of things I was going through and going on in my life. But now I can't…”* (Chloe, 19-year-old woman). Although young people wanted to discuss their feelings about cancer, they felt this was something which would complicate home life and instead avoided communication. This intensified feelings of loneliness for the young people in this study.

#### Subtheme 2: feeling excluded from important medical information

Young people felt excluded from important medical information, which reinforced ideas that they were alone in dealing with parental cancer. In one case, this was because there were younger children in the household who not aware of the cancer diagnosis: *“if we were having a conversation… then my little brother would walk in… we'd have to stop talking about it so sometimes we just wouldn't pick up where we left off”* (Eve, 19-year-old woman). Such circumstances made it logistically challenging for the young person to ask questions about their parent’s illness and limited discussion of cancer at home.

Some participants felt that their parent purposefully withheld medical information in an effort to protect them: *“They don't want to… worry… us with such a problem, which I understand”* (Ben, 19-year-old man). Although the aim of withholding information was not to worry the young person, this led to loneliness at home. This mirrors how young people also avoid communication at home, so they do not burden their parent, suggesting a reciprocal concealment within the parent–child relationship when a parent has cancer. The lack of family conversation about cancer meant some young people were unsure of their parents’ condition during the interview, with young people reporting: *“I’m not like very involved in it”* (Amelia, 19-year-old woman)*.*

Other young people were frustrated at being excluded from information and attempted to obtain it themselves from the internet or by talking to other adults *“I know she speaks… to some of her friends … I just get some self-information from them that she really wouldn't want to share with me”* (Ethan, 18-year-old man). Many adolescents in this study had a developed understanding of cancer and were able obtain their own information on it. Despite this, their parents often decided to shield them from conversations about cancer. Withholding medical information contributed to feelings of loneliness in young people.

#### Subtheme 3: grieving the loss of who their parent was before cancer

Young people deeply missed who their parent was before their diagnosis and reported that their parent had changed physically, mentally, and emotionally because of cancer. This was a barrier to the young person being able to connect with their parent who now seemed unfamiliar to them. Psychologically, the parent in some cases struggled to cope with their diagnosis and behaved differently than usual: “*…she doesn't really like do things the way she used to do…now it's just opposite…Everything has totally changed”* (Chloe, 19-year-old woman). This created a feeling of grief in the young people, and a longing for the personality their parent had before they were diagnosed.

Young people noted appearance changes in their parent due to their treatment which made their parent appear unfamiliar to them: *“…my mum is getting some drip… my mum's skin has changed…everything about how her has changed”* (Chloe, 19-year-old woman). These physical changes were unnerving for young people and created a barrier to connecting with their parent.

Although some parents tried to maintain normalcy for their child, often the young person knew something was wrong:* “Whenever I come home… I know behind the smile… it's not really an in-depth smile”* (Ethan, 18-year-old man). This suggests that young people are very perceptive to small changes when a parent is unwell. One young person stated that they were lonely because their parent was no longer mentally present due to their cancer: *“he has changed a lot… he's thinking more about his diagnosis rather than… more enjoyable times he would have been having with family”* (Callum, 19-year-old man). This created a feeling of the parent’s emotional presence being gone, leading to a paradoxical loneliness of missing someone who is physically present. Parents who were once a reliable source of comfort to the young person had experienced profound changes as a result of cancer, leaving the young person feeling their parent was unfamiliar.

#### Subtheme 4: normal family life shrinks around cancer

Young people noticed that normal family life shrunk around the parent’s cancer diagnosis. Suddenly there was a new and strange ‘normal’ which now included hospital appointments and medical treatments: *“hospital visits…become very normal”* (Callum, 19-year-old man). This disrupted everyday family life. In some cases hospitalisations went on for long periods of time, which was particularly destabilising for young people: “*… he spent a lot of months in the hospital.”* (Helen, 18-year-old woman).

There was a significant shift in family functioning whereby the parent had to now focus on their recovery. This led to situations where the young person felt unrecognised as being impacted by parental cancer. Several participants reported they were not receiving any emotional connection from their parent: “*most times I just feel my mom is not giving me the attention because her diagnosis has cost her a whole lot of her time and her attention”* (Chloe, 19-year-old woman).

Cancer can monopolise the parent’s time leading to young people feeling lonely and abandoned while cancer becomes the priority at home. Participants felt a sense of grief and injustice that their family life had irrevocably changed: *“All his attention has been focused on his recovery and there’s no more time for personal discussions”* (Callum, 19-years-old). In some cases the parent needed time alone to physically recover.This was upsetting for the young person who felt pushed away: *“She just wants to be alone most times she just aches and tells me she wants to be alone.”* (Ethan, 18-year-old man).

In cases where the parent did not need to be alone, their unstable emotions made the young person uncomfortable and created distance in the relationship *“she's always crying…she's making us very uncomfortable…… it's really difficult for me”* (Chloe, 19-year-old woman). This impacted the quality of the parent–child relationship for some young people, which intensified feelings of loneliness: “*I'm no longer being able to enjoy the relationship I have with her anymore”* (Ethan, 18-year-old man). Regular family functioning shrinks around a cancer diagnosis which intensifies loneliness for young people.

### Theme 3: feelings of social loneliness among peers when a parent has cancer

This theme reflects how young people experiencing parental cancer often experience social loneliness among their peers. This can stem from young people deciding to keep their parent’s diagnosis private from peers. Young people also report frustrations when peers have upsetting reactions to finding out about parental cancer. Even in cases where young people are keen to socialise with their peers, their capacity to do so is often limited by increased domestic responsibilities while their parent is ill.

#### Subtheme 1: hiding the pain of parental cancer from friends

Many young people reported that they decided to withhold information about parental cancer from their friends. This decision to selectively withdraw from some peers meant that in many cases, the young people were forced to process parental cancer alone, which further compounded their experiences of loneliness.

There were many reasons young people choose not to share with friends. For some participants, they simply considered themselves private and felt it was not appropriate to discuss parental cancer with their friends: *“Something’s going on in my life, that's even very personal for me to discuss”* (Helen, 18-year-old woman). The decision to maintain privacy might be related to a feeling that peers will not understand their experience of parental cancer.

One young person decided not to share about their parent’s cancer as it might impact the emotional climate with friends: *“I wouldn't want…to tell.. because I wouldn't want to…put a damper on moods…”* (Eve 19-year-old woman). Whilst the choice to shield friends from their parent’s diagnosis might help young people maintain a normal social life in the short term, it also can create an emotional loneliness where young people are unable to confide in peers.

In other instances, the young person avoided speaking to peers about parental cancer because they felt hopeless and didn’t see social support as something which could ‘solve’ parental cancer: “*I don't even really have friends anymore because I feel like that is genuinely not going to solve my problems”* (Chloe 19-year-old woman). This suggests that young people experiencing parental cancer might perceive confiding in peers as something which is unable to help them and instead they feel safer to withdraw into solitude.

In some cases, friends tried to reach out to provide emotional support to the young person, but this was not always welcome: *“They always miss me. They always like… trying to be there for me. But it's … it's not going to like to do anything… I don't think… I'm going to meet them”* (Chloe 19-year-old woman). Although young people experience loneliness when their parent has cancer, they often do not feel comfortable speaking to peers about cancer, due to feeling fundamentally different from others. This reluctance to confide in peers might also be attributed to fatalistic coping where individuals feel powerless against their circumstances, and instead simply accept them.

#### Subtheme 2: navigating pity and reactions to the word ‘cancer’

Several young people in this study decided to share the news of their parent’s cancer with some close peers, however this was often difficult to disclose, and young people struggled to navigate how others reacted to the news. For some participants, how their peers reacted intensified feelings of anxiety in the young person: *“I knew that it was a mild form of cancer …but when I tell other people their reactions would kind of scare me more”* (Eve, 19-year-old woman). This suggests young people do not always react appropriately to the news, which might dissuade young people from sharing more information about parental cancer.

One young person in this study pointed out that many other young people are uneducated about cancer, and may think exclusively of aggressive forms of cancer when they hear the news: “*when people are growing up they hear cancer… their face… drops… they think of lung cancer and skin cancer and dying and old people dying.”* (Eve, 19-year-old woman) This can lead to disproportionate reactions from friends which might make young people feel misunderstood and reinforce feeling lonely whilst navigating parental cancer.

Young people also struggled when dealing with sympathetic responses from their peers which felt dismissive or ingenuine: *“it seems like it's just empathy and sympathy.”* (Ethan, 18-year-old man). Such responses were upsetting for young people and highlighted to them that others perceive their parent as helpless: *“… they are being empathetic… I often don't really like it because it … reminds me of her condition and how helpless she could be… any form of reminder… it's a threat to me and it…leaves me in a sombre mood”* (Ethan, 18-year-old man). Inappropriate or pitying reactions from peers likely make young people feel more alone in their experiences of parental cancer and can deter them from disclosing this information to others.

#### Subtheme 3: caring responsibilities limit time to socialise

Participants were less free to socialise when their parent was unwell and came across logistical barriers to spending time outside of the home. When a parent is diagnosed with cancer, domestic obligations a young person is tasked with can vary from small additional responsibilities to full-time caregiving duties for the parent. These additional responsibilities can isolate the young person at home while their friends are freer to socialise. The participants reported that they had to prioritise helping more at home due to their parent experiencing the physical impact of cancer. One young person noted: *“I'd try to help around the house… talk to my mum…more…make sure that she was doing okay after going through a lot of pain…she couldn't really walk around freely”* (Eve, 19-year-old woman).

Another young person noted socialising would not have been feasible due to domestic obligations: *“My schedule was definitely…different.. in the morning my dad was going to chemo I'd have to look after the dog … I couldn't have left…even if I wanted to”* (Amelia, 19-year-old woman). For another participant, he was tasked with full time caregiving to his father which left little opportunity to spend outside of the home: *“we have just been given caring responsibilities.”* (Callum, 19-year-old man). This reflects not only logistical barriers to socialising with friends but may also cause feelings of emotional loneliness when providing caregiving.

Ultimately friendship became less of a priority for young people when the parent was ill and intensified feelings of loneliness: *“When my mum was getting her treatments… my friends were meeting up I… wouldn't really meet up with them”* (Eve, 19-year-old woman). The pressure to stay home and take on more household responsibilities when a parent has cancer presents difficult barriers to connecting with peers and getting support outside of the family home. This likely contributes towards feelings of loneliness.

### Theme 5: protective barriers against loneliness when a parent has cancer

This theme discusses how protective barriers can shield young people from experiencing loneliness when their parent has cancer. Young people described circumstances and strategies which helped them feel more supported when their parent was unwell. This included confiding in selected peers and seeking support from mental health professionals or extended family members. Some young people also described that they would have valued an opportunity to discuss parental cancer with young people who had also experienced this.Participants highlighted techniques such as distraction based coping and emotional expression-based coping shielded them from loneliness.

#### Subtheme 1: relating to those with similar experiences

Several young people in this study did not know any peers who also had experienced parental cancer. Despite this, participants reported that discussing parental cancer with a peer with similar lived experience would have been valuable. Young people highlighted that a young person with lived experience would have a more developed understanding of how parental cancer impacts a young person compared to their peers without this experience. One participant stated that guidance from a peer with lived experience could have helped her to better understand the impact of parental cancer: *“But if I could find someone who.. was in the same place… it would have been really more understandable.”* (Helen, 18-years-old).

Several young people in this study had a peer with similar lived experiences and they found this person easier to confide in than other young people. One young person noted that conversations with those with lived experience had a lighter tone: *“It wasn't as serious of a conversation. It was kind of more like a joke… it was more lighthearted”.* (Eve, 19-year-old woman). Conversations with peers with lived experience create an opportunity to connect with a friend on a deeper level and speak about cancer openly without the constraints of social stigma.

One young person who took part in this study received support from peers with lived experience in a more formalised way, as part of a young person support group: “*I met a group of kids who… had either a mum or dad who was suffering from cancer… those were the persons I felt comfortable… talking about this whole situation with.”* (Helen, 18-year-old woman). Beyond providing the space to discuss cancer, the group also focused on recreational activities as a respite from parental cancer: *“sometimes we just have fun together or just talk about things that we didn't really share with other friends who didn't understand.”* (Helen, 18-year-old woman).

Although other participants had not accessed formal support groups, they agreed that they would value taking part in a peer support group: *“It would be very, very helpful.. I want to speak with someone that is going through what I'm going through..”* (Chloe, 19-year-old woman). Connecting with those with similar lived experiences is an essential protective shield against experiencing loneliness when a parent has cancer.

#### Subtheme 2: socialising with friends

Although many young people in this study initially hid parental cancer from their friends and peers at school, several participants did eventually confide in close peers and found this helpful. The opportunity to connect with and socialise with friends was essential to shield young people from feeling alone when their parent had cancer. One young person noted that although she kept her parent’s diagnosis a secret from most peers at school, she felt it was useful to inform her very close friends: “*I was able to tell my close friends, my really best friends about it.”* (Eve, 19-year-old woman).

In a few cases, some friends who were informed of parental cancer were emotionally responsive and adapted their support by visiting the young person in their home. This circumvents barriers to socialising such as not wanting to leave the ill parent unattended, while also allowing for time to connect with a peer. *“They often visit me on weekends…when I'm less busy and try to make me enjoy the moment.”* (Callum, 19-year-old man).

Several of the participants in this study had recently transitioned to attending university and spoke positively of their experience of making new friends. Participants reported that it was easy to chat to new people during lectures or tutorials: “*Well, I meet people during lecture hours and that has been really so helpful for me”* (Callum, 19-year-old man). It was easier to discuss parental cancer with university aged peers due to their developmental and emotional maturity: *“with my friends I've made at uni, I have no problem telling them. They do get a little bit worried, but I think they're older as well. We're all older now”* (Eve, 19-year-old woman). This finding suggests that conversations about parental cancer are more easily facilitated at university, rather than when the participants were at school.

#### Subtheme 3: getting support from trusted adults

Young people found that support from trusted adults in their life shielded them from loneliness when their parent was ill. It was important for the young people to have at least one grown up who they could confide in about parental cancer. In some cases this was a professional from Child and Adolescent Mental Health Services or a school counsellor who was able to provide mental health support: *“I did end up having to talk to a guidance counsellor… that really helped…just being able to talk about it… voice …concerns I had.”* (Eve, 19-year-old woman). This allowed the young person to speak candidly about the impact of parental cancer without any concern of upsetting their parents.

For some young people the support they received from a trusted adult was more informal and was provided by a family member. One young person reported that even when their parent was ill, they remained emotionally available to them despite their illness: *“My dad is always there for me to talk about it”* (Helen, 18-year-old woman). One young person found support from an uncle who was able to signpost them to mental health services *“…my uncle, and I tried to explain everything to him, and he was able to assist me and from there, I was able to share it”* (Liam, 18-year-old man)*.* Young people found it helpful to confide in a trusted grown-up and while this person often differed across participants, it was usually an adult who was able to hold emotional space for the young person.

#### Subtheme 4: emotion regulation techniques

Young people reported a range techniques they utilised to emotionally regulate themselves when they felt lonely. Some young people reported using distraction-based coping which aimed to distract them from ruminating thoughts. For example, video games were to escape difficult thoughts and emotions caused by parental cancer; *“If I'm to engage in activities… that are engaging like play video games it would be much more distracting for me…I might not really want to follow on the thoughts”* (Ethan, 18-year-old man).

For other young people, using behavioural activation techniques such as going for a walk was helpful: *“I can just go out, I'll go around a little bit and then from there I can just…refresh myself”* (Liam, 18-year-old man)*.* This environmental change allowed the young person to take some space from parental cancer and maintain some normalcy outside of home life.

Some participants confronted their emotions and thoughts about loneliness and parental cancer using emotional expression strategies: *“Yeah like I have got a journal as well, so I always write stuff down which really helps”* (Amelia, 19-year-old woman). This allowed the young person to process their ruminating thoughts and provided a private outlet for expressing how parental cancer was impacting them. Although techniques differed across participants, all young people who took part in this study had at least one technique could help them emotionally regulate.

## Discussion

This is the first qualitative study to give a dedicated focus to adolescent loneliness when a parent has cancer. Many of the findings overlap with the findings of our qualitative JBI systematic review on adolescent loneliness [31]. Both studies found that young people experiencing parental cancer experience limited peer support due to feeling different from their friends and must prioritise domestic responsibilities over socialising. Similarly, both papers indicated that young people struggled to manage negative emotions alone and felt lonely within their family life due to being excluded from medical information and avoiding communicating with parents for fear of upsetting them. While some peers can occasionally be supportive, a consistent finding across both papers is that young people would rather discuss their experiences of parental cancer with others who have similar lived experiences. Contrastingly to our review [31], the current study provides a deeper conceptualisation of how young people describe and embody the experience of loneliness when their parent is ill. For example, the current study sheds light on experiences of existential loneliness which have not been previously reported in this population. Together, the current study and our review [31] indicate that adolescents experiencing parental cancer are particularly vulnerable to experiencing loneliness.

### Facets of intrapersonal loneliness

Participants in this study focused on describing how they conceptualised and experienced loneliness when their parent had cancer. This occurred on several levels including temporal loneliness; affective loneliness; relational loneliness; existential loneliness and feeling like a changed person as a result of parental cancer. participants drew comparisons to negative experiences to describe their loneliness, including loneliness as a feeling of ‘loss’. A recent systematic review [30] of loneliness across the lifespan also reports that people can experience loneliness as a feeling of loss or disconnection from others. This feeling of disconnection when experiencing loneliness has also been reported in adolescent samples specifically [50]. This suggests there is some overlap between how adolescents experience loneliness when a parent has cancer, and how it is experienced by adolescents more generally.

Several unique experiences of loneliness emerged in this population which link closely to experiences of loneliness among clinical health populations, such as existential loneliness. Young people discussed feeling separate from others. A review of existential loneliness [2]describes the phenomenon as perceiving oneself as intrinsically separated from other people and the universe [2]. Existential loneliness is typically researched within end-of-life populations [16]including patients with advanced cancer [42], as confrontation with death is thought to intensify an awareness of one’s intrinsic separateness from others. All participants in this study reported that their parent had or was receiving treatment for cancer with the intention of eliminating the disease. Despite this, young people still worried their parent may pass away, and many participants found it difficult to understand why cancer had happened to their family. This finding suggests that existential loneliness is also experienced by close persons to the cancer patient and are not exclusive to end of life experiences. The experience of existential loneliness in this study contrasts to presentations of existential loneliness in general youth populations. Typically this is described as feeling lost in life, struggling with low self-esteem and low confidence [23]. Issues of with low self-esteem and low confidence were not reported within the current sample. This suggests that existential loneliness when a parent had cancer is tied directly to the challenges of cancer specifically [23].

Young people in the current study felt like a changed person as a result of their parent having cancer, and this contributed toward their feelings of loneliness. Similarly, young people noticed that their parent had changed as a result of cancer and this was distressing This finding aligns closely with literature on cancer-related loneliness in adults [38] which report accounts of feeling like a changed person as a result of cancer. Patients noted difficulty integrating their experience of cancer into their current identity and felt a layer of separation compared to the person they were prior to treatment [38]. Young people in the current study reported similar feelings of identity change. Contrastingly to adult cancer survivors experiencing loneliness [38], young people felt different as a result of having to become more mature to deal with their parent’s illness. Young people also felt their identity had changed because their life had been disrupted by cancer and no longer fitted their expectations. This is known as biological disruption [10] and it has been linked to loneliness in older adults [32]. Older participants [32]struggle to accept how their life had changed in unexpected ways and found this to exacerbate experiences of loneliness. Similarly, young people in the current study reported that their lives were disrupted by parental cancer, and their everyday experience of normality was altered. Cancer not only changes the life of the individual diagnosed, but also the lives of their children.

Young people in this study discussed experiences of affective loneliness and framed loneliness as a feeling of emotional distress. In one case, a young person described loneliness as suicidal feelings. Although it is challenging to comment on the directionality of the relationship between depression and loneliness, it is common across qualitative interview studies for individuals to discuss low mood as a central component of An analysis of conversations with ChildLine counsellors suggests young people characterise the emotional side of loneliness as “sadness” [51]. Young people also discussed feelings of anxiety throughout the interviews. Although rumination is thought to be a key feature of loneliness [54], worry is also part of the broader experience of a parent having cancer [53]. Some young people specifically mentioned dealing with anxiety attacks or ruminating alone suggesting anxiety might be intensified by loneliness. This aligns to our review evidence [31]which also reported that adolescents experiencing parental cancer dealt with overwhelming negative emotions alone. It is evident that feelings of low mood and worry are central to the experience of loneliness when a parent has cancer.

### Loneliness in family life

Participants reported experiences of loneliness within family life due to their parent’s cancer. Young people limited their communication at home to avoid burdening the ill parent. A qualitative study of children and young people with a parent with a life-limiting illness indicates that young people feel a pressure to look after their family and avoid causing any stress at home when their parent is sick [29]. While this attitude might help support family functioning when a parent has cancer, it can lead to the parentification of the young person whereby the child is responsible for supporting the adult [39]. The current study demonstrated instances of parentification where the young person prioritised their parent’s comfort and emotional state above their own needs. This reinforced feelings of loneliness.

Young people felt isolated from important medical information about their parents’ cancer. Literature on parental cancer has previously identified that young people can feel lonely as a result of unmet informational needs and constrained communication with their parents about cancer [26, 31]. This is also evident in populations where the parent has a mental illness. Young people describe a paradoxical silence at home where the mental illness is not discussed even though it deeply impacts every aspect of life [44]. Similar feelings were reported in the current study, young people felt deeply impacted by cancer but lacked specific information on the diagnosis and treatment. Feeling uninformed about cancer was isolating and intensified feelings of loneliness.

Young people in this study reported feelings of grief and loss. Young people missed who their parent was before cancer and found their usual family life now had to shrink around the diagnosis. It has been reported that cancer changes the dynamics of home life for young people, contributing toward feelings of loneliness [31]. However, there is a lack of literature which discusses grieving who a parent was prior to their cancer diagnosis. Within the wider literature, there is some information on adult cancer caregivers reporting experiences of anticipatory grief prior to bereavement which caused elevated levels of loneliness [20]. All participants in this study had a parent diagnosed with a curable rather than an advanced cancer. This suggests that it is unlikely that the feeling of loss they described can be attributed to anticipatory grief. It is possible that the young people were experiencing ambiguous loss, described as a sense of loss when a loved one is psychologically absent but physically present [3]. Young people felt that although their parent was present, they had changed physically and emotionally and were often distracted by their diagnosis. It is likely that young people’s experiences of loneliness when their parent has cancer might be further intensified by ambiguous loss.

### Loneliness in peer groups

Young people experienced loneliness among peers and decided in most cases to hide parental cancer from friends A lack of support from peers is the most reported unmet according to young people who have a parent with cancer [53], thereby making it an unsurprising source of loneliness for this group of young people. Participants in the current study had a very different life from peers due to their unique circumstances of parental cancer, which likely intensified their experiences of loneliness. Young people themselves who are chronically ill report that feeling different from peers can cause feelings of isolation and loneliness [21]. Young people experiencing parental cancer also compared their lives to peers in a similar manner, feeling they were intrinsically different to their friends. This suggests that social impacts of chronic illness extend not just to the person diagnosed, but also their close family. Both these cohorts of youth engage in an upward social comparison with peers which can intensify feelings of social isolation and loneliness.

It is common for young people experiencing parental cancer to report not discussing their parent’s illness with peers and rejecting peer support when it is offered [31]. This is consistent with the findings from the current study. This finding is a departure from more general experiences of youth loneliness among peers as it is rooted in specific challenges connected to parental cancer. Young people experiencing loneliness among peers more typically attribute loneliness to issues of interpersonal conflict, exclusion and bullying [46]. The participants in this study provided accounts of loneliness within their peer groups which links more closely to how loneliness is experienced by cancer patients themselves. Patients report finding friends uncomfortable discussing cancer and often decide to withhold medical information from them [1]. It is possible this behaviour around friends is modelled to young people by the diagnosed parent and impacts how the young person decides to discuss cancer with their own peers. The interpersonal loneliness adolescents experience when a parent has cancer is unique to their parent’s illness and may differ from typical presentations of youth loneliness.

### Protective barriers against loneliness

Young people in the current study reported that it was comforting to speak with young people who have lived experience of parental cancer and that socialising with close peers was also beneficial. Findings from a peer support group for young people experiencing parental cancer indicated a reduction in young people's self‐reported loneliness [45]. These findings are also consistent with our systematic review evidence which highlighting that friends, especially those with lived experience of parental cancer, can be a vital source of support Mckeown et al. [31]. Two young people in this study were first year university students, and they described new friends as a source of protection against loneliness. This is an interesting finding as beginning university is believed to be a challenging life transition which is associated loneliness for young people [48]. The young people in this study who had just begun university found their university aged friends more able to process and handle information about parental cancer than school aged peers were. This finding highlights another way in which loneliness when a parent is experiencing cancer may be unique and differ from youth loneliness more generally.

Young people also reported that it was helpful to speak to a trusted adult, in some cases this was an extended family member.. With this in mind, extended family members may also be worth targeting when considering family-centred support for a young person whose parent is diagnosed with cancer. Other young people sought formalised treatment and support from mental health professionals or counsellors to deal with loneliness. This reflects national patterns of young people seeking support for loneliness. Recent data indicates that Childline delivered over 4,500 counselling sessions to lonely youth in 2024 [34]. Young people also found ways to support themselves through loneliness when a parent has cancer using a range of emotional regulation strategies. Some young people took an emotional-expression approach to coping with journalling while other young people favoured avoidant strategies such distraction. A systematic review [13] on the association between loneliness and coping strategies indicates that problem-focused coping styles are associated with lower levels of loneliness while emotion-focused coping styles is associated with higher levels of loneliness [13]. This is interesting as few young people in this study reported using problem-focused coping to deal with loneliness when their parent had cancer, such as reaching out to friends or joining a club. This may have been due to the fact they were less able to go out and socialise when their parent was unwell, meaning problem-focused coping may be less accessible to this particular cohort of lonely youth.

### Strengths

This study is the first known qualitative study internationally which has specifically focused on adolescent loneliness when a parent has cancer. It has provided a unique insight as to how a specific subgroup of youth describe their lived experience of loneliness. The use of qualitative interviews supported a deep and nuanced understanding of the participant’s subjective lived experience. This study aligned with key research priorities outlined by our qualitative systematic review [31] on adolescent loneliness when a parent has cancer which called for this cohort of young people to be interviewed directly. This study also directly addressed research priorities highlighted in by the UK governments *Tackling Loneliness Evidence Review* (UK Government, 2018), which emphasised a need for youth loneliness research to consider small subgroups of youth and to prioritise in-depth qualitative work to explore the meaning of loneliness. The findings of the current study have given a voice to lonely young people experiencing parental cancer and contributed toward our conceptual understanding of loneliness in this cohort. The present study further addresses key research priorities highlighted by *The James Lind Alliance Priority Setting Partnership* (NIHR, 2018) which indicated a need to explore the impact of parental cancer and how best to support young people when their parent has cancer. The current study shows that the impact of parental cancer can cause deep feelings of loneliness for young people and highlighted protective factors which might support this vulnerable cohort of youth.

### Limitations

The sample of this study included experiences of Black and White young people across England, Scotland and Northern Ireland. It should however be noted that there were no participants from other ethnic backgrounds such as South Asian, East Asian or Middle Eastern communities. Given that there are many individuals from these ethnic backgrounds within the UK, it must be noted that our findings may not extend to all young people in the UK experiencing loneliness and parental cancer. Although the current study was open to young people aged 10 to 19 years old, all the participants who took part were aged 18–19 years old. As a result, the findings of the current study may not be applicable to younger adolescents who may have a different experience of loneliness when a parent has cancer.

### Future research

Although this study aimed to investigate adolescent loneliness among 10- to 19-year-olds, the final sample included only 18- and 19-year-old adolescents. Future research should consider interviewing younger children on loneliness when a parent has cancer who may experience loneliness differently due to their developmental stage. Although systematic review evidence indicates that there are interventions which aim to support young people when a parent has cancer [25], it should be noted that there are currently no evidence-based interventions to specifically ameliorate loneliness in young people when their parent has cancer. Future research should consider developing an intervention to support this vulnerable cohort of young people specifically experiencing loneliness. With this in mind, it is also necessary for more in-depth longitudinal research on how adolescent loneliness develops across the parental cancer continuum, with the view of future interventions targeting different stages of the parental cancer trajectory.

### Clinical implications

Young people in this study were profoundly impacted by their parent’s cancer but did not receive adequate communication about their parents’ condition. This intensified experiences of loneliness for young people. It is essential for healthcare teams to screen patients early on to establish if they have dependent young people who may be impacted by parental cancer. This would provide an opportunity for healthcare professionals to explain to parents that open family communication is protective to young people. Early detection of dependent young people also provides an opportunity for healthcare teams to signpost young people to dedicated support services. Services across the UK such as Hope Support Services provide online peer support groups to help young people connect to those with similar experiences of family illnesses including cancer (Hope Support Services, n.d.). With young people in the current study reporting that talking to peers with similar life experiences is beneficial, it is vital families are signposted to services which can provide this opportunity.

## Conclusion

Adolescents experiencing parental cancer report experiences of intrapersonal loneliness and interpersonal loneliness across their peer group and family life. This loneliness was related to negative emotional experiences such as feelings of depression and anxiety. Potential key support for young people experiencing loneliness when a parent has cancer might include support from friends with lived experience and support from trusted adults. By centring the voice of young people experiencing loneliness and parental cancer, current services that support families with cancer can be better informed as to how loneliness may impact dependent young people. This study also provides a strong foundation to upon which interventions can be built. Despite the fact that the literature base on adolescent loneliness when a parent is experiencing cancer is limited, it is clear from this study that loneliness is central to their experience.

## Supplementary Information

Supplementary Material 1.

## Data Availability

The interview data which supports this manuscript is available upon reasonable request to corresponding author LM.

## References

[1] Adams RN, Mosher CE, Abonour R, Robertson MJ, Champion VL, Kroenke K. Cognitive and situational precipitants of cancer patients’ loneliness: a qualitative analysis. InOncology Nursing Forum. 2016;43(2):156. 10.1188/16.ONF.156-163 PMC4899046 26906127

[2] Bolmsjö I, Tengland PA, Rämgård M. Existential loneliness: an attempt at an analysis of the concept and the phenomenon. Nurs Ethics. 2019;26(5):1310–25. 29471724 10.1177/0969733017748480

[3] Boss P. The trauma and complicated grief of ambiguous loss. Pastoral Psychol. 2010;59(2):137–45.

[4] Braun V, Clarke V. Using thematic analysis in psychology. Qualitative research in psychology. 2006;3(2):77–101.

[5] Braun V, Clarke V. What can “thematic analysis” offer health and wellbeing researchers? International journal of qualitative studies on health and well-being. 2014;9(1):26152. 25326092 10.3402/qhw.v9.26152 PMC4201665

[6] Braun V, Clarke V. Reflecting on reflexive thematic analysis. Qualitative research in sport, exercise and health. 2019;11(4):589–97.

[7] Braun V, Clarke V. One size fits all? What counts as quality practice in (reflexive) thematic analysis? Qual Res Psychol. 2021;18(3):328–52.

[8] Braun V, Clarke V. Conceptual and design thinking for thematic analysis. Qualitative psychology. 2022;9(1).

[9] Braun V, Clarke V. Supporting best practice in reflexive thematic analysis reporting in Palliative Medicine: a review of published research and introduction to the Reflexive Thematic Analysis Reporting Guidelines (RTARG). Palliat Med. 2024;38(6):608–16. 38469804 10.1177/02692163241234800 PMC11157981

[10] Bury M. Chronic illness as biographical disruption. Sociol Health Illn. 1982;4(2):167–82. 10260456 10.1111/1467-9566.ep11339939

[11] Cena E, Brooks J, Day W, Goodman S, Rousaki A, Ruby-Granger V, et al. Quality criteria: general and specific guidelines for qualitative approaches in psychology research. A concise guide for novice researchers and reviewers. Int J Qual Methods. 2024;23:16094069241282844.

[12] Deckx L, van den Akker M, Buntinx F. Risk factors for loneliness in patients with cancer: a systematic literature review and meta-analysis. Eur J Oncol Nurs. 2014;18(5):466–77. 24993076 10.1016/j.ejon.2014.05.002

[13] Deckx L, van den Akker M, Buntinx F, van Driel M. A systematic literature review on the association between loneliness and coping strategies. Psychol Health Med. 2018;23(8):899–916. 29533084 10.1080/13548506.2018.1446096

[14] Deckx L, van den Akker M, van Driel M, Bulens P, van Abbema D, Tjan‐Heijnen V, et al. Loneliness in patients with cancer: the first year after cancer diagnosis. Psycho-Oncol. 2015;24(11):1521–8. 10.1002/pon.3818 25914244

[15] Dwyer SC, Buckle JL. The space between: on being an insider-outsider in qualitative research. Int J Qual Methods. 2009;8(1):54–63.

[16] Ettema EJ, Derksen LD, van Leeuwen E. Existential loneliness and end-of-life care: a systematic review. Theor Med Bioeth. 2010;31(2):141–69. 20440564 10.1007/s11017-010-9141-1 PMC2866502

[17] Fletcher AJ. Applying critical realism in qualitative research: methodology meets method. Int J Soc Res Methodol. 2017;20(2):181–94.

[18] Gazendam-Donofrio SM, Hoekstra HJ, van der Graaf WT, van de Wiel HB, Visser A, Huizinga GA, et al. Adolescents’ emotional reactions to parental cancer: effect on emotional and behavioral problems. J Pediatr Psychol. 2011;36(3):346–59. 20929959 10.1093/jpepsy/jsq090

[19] Goosby BJ, Bellatorre A, Walsemann KM, Cheadle JE. Adolescent loneliness and health in early adulthood. Sociol Inquiry. 2013;83(4):505–36. 10.1111/soin.12018 PMC3810978 24187387

[20] Gray TF, Azizoddin DR, Nersesian PV. Loneliness among cancer caregivers: a narrative review. Palliat Support Care. 2020;18(3):359–67. 31581964 10.1017/S1478951519000804

[21] Heaton J. Use of social comparisons in interviews about young adults’ experiences of chronic illness. Qual Health Res. 2015;25(3):336–47. 25281241 10.1177/1049732314553122 PMC4361472

[22] Heinrich LM, Gullone E. The clinical significance of loneliness: a literature review. Clin Psychol Rev. 2006;26(6):695–718. 16952717 10.1016/j.cpr.2006.04.002

[23] Hemberg J, Östman L, Korzhina Y, Groundstroem H, Nyström L, Nyman-Kurkiala P. Loneliness as experienced by adolescents and young adults: an explorative qualitative study. Int J Adolesc Youth. 2022;27(1):362–84.

[24] Huizinga GA, Visser A, van der Graaf WT, Hoekstra HJ, Hoekstra-Weebers JEM. The quality of communication between parents and adolescent children in the case of parental cancer. Ann Oncol. 2005;16(12):1956–61. 16143592 10.1093/annonc/mdi395

[25] Inhestern L, Haller AC, Wlodarczyk O, Bergelt C. Psychosocial interventions for families with parental cancer and barriers and facilitators to implementation and use–a systematic review. PLoS ONE. 2016;11(6):e0156967. 10.1371/journal.pone.0156967 PMC4898703 27276079

[26] Karlsson E, Andersson K, Ahlström BH. Loneliness despite the presence of others–adolescents' experiences of having a parent who becomes ill with cancer. Eur J Oncol Nurs. 2013;17(6):697–703. 10.1016/j.ejon.2013.09.005 24183584

[27] Loades ME, Chatburn E, Higson-Sweeney N, Reynolds S, Shafran R, Brigden A, et al. Rapid systematic review: the impact of social isolation and loneliness on the mental health of children and adolescents in the context of COVID-19. J Am Acad Child Adolesc Psychiatry. 2020;59(11):1218–39. 32504808 10.1016/j.jaac.2020.05.009 PMC7267797

[28] Malterud K, Siersma VD, Guassora AD. Sample size in qualitative interview studies: guided by information power. Qual Health Res. 2016;26(13):1753–60. 26613970 10.1177/1049732315617444

[29] Mansfield L, Victor C, Meads C, Daykin N, Tomlinson A, Lane J, et al. A conceptual review of loneliness in adults: qualitative evidence synthesis. Int J Environ Res Public Health. 2021;18(21):11522. 34770035 10.3390/ijerph182111522 PMC8582800

[30] McKenna-Plumley PE, Turner RN, Yang K, Groarke JM. Experiences of loneliness across the lifespan: a systematic review and thematic synthesis of qualitative studies. Int J Qual Stud Health Well-being. 2023;18(1):2223868. 37327403 10.1080/17482631.2023.2223868 PMC10281437

[31] Mckeown L, Campbell K, Dempster M, Groarke J, Graham-Wisener L. Adolescent loneliness when a parent has cancer: a qualitative systematic review. Psycho-Oncol. 2025;34(4):e70148. 10.1002/pon.70148 PMC11979319 40200422

[32] Morgan DJ, Burholt V. Loneliness as a biographical disruption—theoretical implications for understanding changes in loneliness. J Gerontol Ser B. 2020;75(9):2029–39. 10.1093/geronb/gbaa097 PMC7566959 32812040

[33] National Institute for Health and Care Research & James Lind Alliance. (N.D). Living with and beyond cancer: top 10 priorities. NIHR James Lind Alliance. 2018. https://www.jla.nihr.ac.uk/priority-setting-partnerships/living-with-and-beyond-cancer#tab-top-10-priorities.

[34] NSPCC. Childline prepares for a rise in children reaching out about loneliness this summer. 2025. https://www.nspcc.org.uk/about-us/news-opinion/2025/childline-prepares-for-rise-in-children-reaching-out-about-loneliness-this-summer/.

[35] Osborn T. The psychosocial impact of parental cancer on children and adolescents: a systematic review. Psycho‐Oncol J Psychol Soc Behav Dimensions Cancer. 2007;16(2):101–26. 10.1002/pon.1113 17273987

[36] Perlman D, Peplau LA. Toward a social psychology of loneliness. Pers Relat. 1981;3:31–56.

[37] Qualter P, Vanhalst J, Harris R, Van Roekel E, Lodder G, Bangee M, et al. Loneliness across the life span. Perspect Psychol Sci. 2015;10(2):250–64. 25910393 10.1177/1745691615568999

[38] Raque-Bogdan TL, Lamphere B, Kostiuk M, Gissen M, Beranek M. Unpacking the layers: a meta-ethnography of cancer survivors’ loneliness. J Cancer Surviv. 2019;13(1):21–33. 30414079 10.1007/s11764-018-0724-6

[39] Schorr S, Goldner L. “Like stepping on glass”: A theoretical model to understand the emotional experience of childhood parentification. Fam Relat. 2023;72(5):3029–48.

[40] Shah BK, Armaly J, Swieter E. Impact of parental cancer on children. Anticancer Res. 2017;37(8):4025–8. 28739684 10.21873/anticanres.11787

[41] Tackling loneliness evidence review: executive summary. GOV.UK. 2023. https://www.gov.uk/government/publications/tackling-loneliness-evidence-review/tackling-loneliness-evidence-review-summary-report.

[42] Tarbi EC, Meghani SH. Existential experience in adults with advanced cancer: a concept analysis. Nurs Outlook. 2019;67(5):540. 31040052 10.1016/j.outlook.2019.03.006 PMC6764914

[43] Tomova L, Andrews JL, Blakemore SJ. The importance of belonging and the avoidance of social risk taking in adolescence. Dev Rev. 2021;61:100981.

[44] Trondsen MV. Living with a mentally ill parent: Exploring adolescents’ experiences and perspectives. Qual Health Res. 2012;22(2):174–88. 21873281 10.1177/1049732311420736

[45] Tucker AR, Sugerman D, Zelov R. On Belay: Providing connection, support, and empowerment to children who have a parent with cancer. J Exp Educ. 2013;36(2):93–105.

[46] Turner S, Fulop A, Woodcock KA. Loneliness: adolescents’ perspectives on what causes it, and ways youth services can prevent it. Child Youth Serv Rev. 2024;157:107442.

[47] UK Government Department for Culture, Media and Sport. Loneliness annual report: the fourth year. GOV.UK. 2023. https://www.gov.uk/government/publications/loneliness-annual-report-the-fourth-year.

[48] Vasileiou K, Barnett J, Barreto M, Vines J, Atkinson M, Long K, et al. Coping with loneliness at university: a qualitative interview study with students in the UK. Mental Health Prevent. 2019;13:21–30.

[49] Vasileiou K, Barnett J, Thorpe S, Young T. Characterising and justifying sample size sufficiency in interview-based studies: systematic analysis of qualitative health research over a 15-year period. BMC Med Res Methodol. 2018;18(1):148. 30463515 10.1186/s12874-018-0594-7 PMC6249736

[50] Verity L, Schellekens T, Adam T, Sillis F, Majorano M, Wigelsworth M, et al. Tell me about loneliness: interviews with young people about what loneliness is and how to cope with it. Int J Environ Res Public Health. 2021;18(22):11904. 34831664 10.3390/ijerph182211904 PMC8621930

[51] Verity L, Yang K, Nowland R, Shankar A, Turnbull M, Qualter P. Loneliness from the adolescent perspective: a qualitative analysis of conversations about loneliness between adolescents and Childline counselors. J Adolesc Res. 2024;39(5):1413–43.

[52] Von Soest T, Luhmann M, Gerstorf D. The development of loneliness through adolescence and young adulthood: its nature, correlates, and midlife outcomes. Dev Psychol. 2020;56(10):1919. 32852969 10.1037/dev0001102

[53] Walczak A, McDonald F, Patterson P, Dobinson K, Allison K. How does parental cancer affect adolescent and young adult offspring? A systematic review. Int J Nurs Stud. 2018;77:54–80. 29035733 10.1016/j.ijnurstu.2017.08.017

[54] Yun RC, Fardghassemi S, Joffe H. Thinking too much: how young people experience rumination in the context of loneliness. J Community Appl Soc Psychol. 2023;33(1):102–22.

