# Supplementary material for: “I just feel alone and by myself”: how adolescents experience loneliness when their parent has cancer
Source: BMC Public Health. 2026 Mar 31;26:1515. doi: 10.1186/s12889-026-27112-x (PMC13159267; doi:10.1186/s12889-026-27112-x)
Supplement: Supplementary file 1 — Supplementary Material 1. [file 12889_2026_27112_MOESM1_ESM.docx]

**16-19 Year Olds Interview Topic Guide**

**Research study:** The Experience of Loneliness During Adolescence When a Parent has Cancer: A Qualitative Interview Study.

**Background Questions**

- Can you tell me about your mum or dad having cancer? (*Prompts – What is it like? How is life different when a parent has cancer?)*

**Loneliness**

- What does being lonely feel like? (*Prompt – when are you lonely, how does it impact you emotionally?,*loneliness is defined as a difference between a person’s desired number of social connections and the actual number of social connections they have.*How would you define loneliness/Do you identify with this definition?*
- Can having a mum/dad with cancer make young people lonely? (*Prompt – where you lonely before or did cancer change things? Why might parental cancer make young people feel lonely?*)
- Is there anything that makes you feel lonely? (*Prompt - Is there anything that makes you feel better*)
- Do you feel understood by other people (*Prompt – do you ever feel separate to others? Do you feel a sense of connectiveness to other people?*)

**Friendship Questions**

- Do you chat to your friends about your parents’ cancer?  (*Prompts – do your friends ask you about cancer? Can you open up to them about cancer?)*
- Has mum/dad having cancer changed things with your friends at all? (*Prompts – what were things with friends like before cancer?*)
- How could a friend help someone who is going through a mum/dad having cancer?  (*Prompt - Is there anything friends do that isn’t helpful?)*
- Do you know any other young person who has a mum or dad with cancer? (P*rompt - If so, what is it like, being friends with them? Is it different than being friends with people who haven’t gone through a mum/dad having cancer?*)

**Summary**

- Is there anything I left out?
- Is there anything else you’d like to share today?
- Okay then, I’ll turn off the recorder now.
- Thank participant.
- Mention sending debrief.
